# Predictive Analysis of Amyotrophic Lateral Sclerosis Progression and Mortality in a Clinic Cohort From Singapore

**DOI:** 10.1002/mus.28416

**Published:** 2025-04-23

**Authors:** Ian Qian Xu, Ling Guo, Jing Xu, Stella Setiawan, Xiao Deng, Yew Long Lo, Josiah Yui Huei Chai, Zachary Simmons, Savitha Ramasamy, Crystal Jing Jing Yeo

**Affiliations:** ^1^ Duke‐NUS Medical School Singapore; ^2^ National Neuroscience Institute Singapore; ^3^ Institute for Infocomm Research (I^2^R), A*STAR Singapore; ^4^ Centre for Quantitative Medicine Duke‐NUS Singapore; ^5^ Department of Neurology Pennsylvania State University Hershey Pennsylvania USA; ^6^ Institute of Molecular and Cell Biology (IMCB) Agency for Science, Technology and Research (A*STAR) Singapore; ^7^ School of Medicine, Medical Sciences and Nutrition University of Aberdeen Aberdeen UK

**Keywords:** ALS registry, machine learning, prognostic factors, survival analysis, survival prediction

## Abstract

**Introduction:**

There is currently no comprehensive Amyotrophic Lateral Sclerosis (ALS) patient database in Singapore comparable to those available in Europe and the United States. We established the Singapore ALS registry (SingALS) to draw meaningful inferences about the ALS population in Singapore through developing statistical and machine learning‐based predictive models.

**Methods:**

The SingALS registry was established through the retrospective collection of demographic, clinical, and laboratory data from 72 ALS patients at Tan Tock Seng Hospital (TTSH) and combining it with demographic and clinical data from 71 patients at Singapore General Hospital (SGH). The SingALS was compared against international ALS registries. Using comparative studies including survival and temporal feature analysis, we identified key factors influencing ALS survival and developed a machine learning model to predict survival outcomes.

**Results:**

Compared to Caucasian‐dominant registries, such as the German Swabia registry, SingALS patients had longer average survival (50.51 vs. 31.0 months), younger age of onset (56.18 vs. 66.6 years), and lower bulbar onset prevalence (20.98% vs. 34.10%). Singaporean males had poorer outcomes compared to females, with a hazard ratio (HR) of 3.12 (*p* = 0.008). Patients who died within 24 months had an earlier need for being bedbound (*p* < 0.004), percutaneous endoscopic gastrostomy (PEG) insertion (*p* = 0.004) and non‐invasive ventilation (NIV) (*p* < 0.001). Machine learning and statistical analysis indicated that a steeper ALSFRS‐R slope, higher alkaline phosphatase (ALP), white blood cell (WBC), absolute neutrophil counts, and creatinine levels are associated with worse mortality.

**Discussion:**

We developed a comprehensive Singaporean ALS registry and identified key factors influencing survival.

**Abbreviations**
ALP: alkaline phosphatase
ALS: amyotrophic lateral sclerosis
ALSFRS‐R score: ALS functional rating scale‐revised
ANOVA: analysis of variance
AUC: area under the receiver operator characteristics curve
AUPRC: area under the precision‐recall curve
BMI: body mass index
ENCALS: European Network for the cure of ALS
HR: hazard ratio
IRB: Institutional Review Board
KM: Kaplan–Meier
NPV: negative predictive value
PEG: percutaneous endoscopic gastrostomy
PLS: primary lateral sclerosis
PPV: positive predictive value
PRO‐ACT: pooled resource open‐access ALS clinical trials
SGH: Singapore General Hospital
SingALS: Singapore ALS
TTSH: Tan Tock Seng Hospital
WBC: white blood cell

## 1 Introduction

ALS is a devastating motor neuron disease with heterogeneous presentation and progression. Existing ALS registries, such as the North American Pooled Resource Open‐Access ALS Clinical Trials [1] (PRO‐ACT) and the European Network for the Cure of ALS [2] (ENCALS), have been used to identify critical prognostic markers. Lower uric acid baseline, lower baseline BMI, bulbar onset, and presence of C9orf72 repeat expansion have been associated with worse survival outcomes [1, 2]. However, research predominantly involves American and European populations, limiting the generalizability of findings across diverse ethnic compositions, particularly within Asian communities. In addition, while predictive models trained on registries such as the ENCALS have been developed for ALS prognosis analysis with good predictive accuracy, their applicability to Asian populations remains uncertain.

Singapore is a highly pluralistic society [3], marked by its diverse and heterogeneous Asian demographics. According to the 2020 population census, the resident population consists of 74.3% Chinese, 13.5% Malays, and 9.0% Indians [4]. The prevalence of motor neuron diseases is 4.33 per 100,000 population in Singapore in 2016, with a total of 243 cases reported [5]. We sought to establish a local ALS registry to enhance our understanding of ALS prognosis in the Asian population. A previous study in SGH identified key predictors for poorer survival outcomes, such as bulbar onset, male gender, and rapid disease progression [6]. However, this study focused on demographic and clinical data and did not collect laboratory information which we had previously found to be important in predicting ALS prognosis [7]. Having a comprehensive ALS registry in Singapore, similar to those found in North America [1] and Europe [2], could promote ALS clinical research and predictive model development in Asia.

This project aims to bridge existing gaps by:

1. Conducting a retrospective study using data collected from two of the three major tertiary medical centers in Singapore. This dataset includes demographic, clinical, and laboratory information from ~60% ALS patients across multiple clinical visits in Singapore. Additionally, the registry is still in the process of being established for prospective studies and will eventually encompass all three major medical centers.
2. Comparing the Singapore registry with international registries to explore geographical and ethnicity differences and similarities across ALS cohorts.
3. Identifying potential prognostic factors affecting ALS survival within the clinic cohort from Singapore.
4. Developing a comprehensive machine learning model to reliably predict mortality and identify the most significant predictive factors among Singapore ALS patients. Specifically, we employed eXtreme Gradient Boosting (XGBoost) [8]—a method that has previously facilitated the creation of an accurate machine learning model for identifying fast‐progressing cases [7].

## 2 Materials and Methods

### 2.1 Study Population and Data Collection

We first reviewed the medical records of 71 patients diagnosed with ALS at SGH, Singapore, between January 2004 and December 2017. The existing SGH ALS database, compiled by Deng et al. [6], includes demographic data and ALS‐specific clinical milestones (Table S1). This study of the SGH database was approved by the SingHealth Centralized Institutional Review Board (IRB) (CIRB Ref: 2014/685/A).

Subsequently, we collected and reviewed the medical records of patients diagnosed with ALS at TTSH, Singapore, from January 2010 to November 2023. The diagnosis of ALS was based on the Gold Coast criteria [9] and confirmatory evaluations by neuromuscular specialists. Patients with ALS‐mimicking conditions [10], such as Primary Lateral Sclerosis (PLS), were excluded. The final TTSH ALS Database comprised demographic features and ALS‐specific clinical milestones derived from 1312 clinical visit records from 72 patients diagnosed with ALS (Table S2). Additionally, we incorporated laboratory data, which were not available in the previous SGH cohort. The collection and analysis of the TTSH ALS database was approved by the SingHealth IRB (CIRB Ref: 2015/2030).

As a result, the merged SingALS database comprised demographic features and ALS‐specific clinical milestones from 143 patients from TTSH and SGH. This represents a big proportion, considering that only 243 reported cases of motor neuron diseases were reported in 2016 [5]. While the sample size may appear limited, it is highly representative of the ALS population in Singapore. Although the National University Hospital (NUH) is another tertiary hospital treating ALS patients in Singapore, we were unaware of a database that could be included in this study.

### 2.2 Comparison With International Databases

We compared the TTSH, SGH, and the combined SingALS database against other notable ALS databases from around the globe [2, 11, 12, 13, 14, 15, 16], encompassing a variety of regions and diverse ethnicities. The databases compared originate from several regions: Germany [11], Italy [12], Europe [2], Malaysia [15], India [14], China [13], and Taiwan [16]. To ensure meaningful comparisons, we selected databases with similar study designs, inclusion criteria, and data collection methodologies. Specifically, this study employed a prevalent cohort for survival analysis, which aligns with prevalent cohort designs used internationally, including ENCALS [2] and registries from Germany [11], Italy [12], Malaysia [15], India [14], China [13], and Taiwan [16]. These cohorts share key characteristics, such as longitudinal follow‐up and standardized clinical assessments, allowing for valid cross‐regional comparisons. As shown in Table 1, the number of recruited patients, gender percentage, age of onset, diagnostic delay, location of onset, time from symptom onset to NIV and PEG, disease duration, and percentage of taking Riluzole were compared across regions. These similarities in study design and patient characteristics support the validity of our comparisons and reinforce the relevance of cross‐population analyzes.

**TABLE 1:** Comparison of ALS clinical features among the international databases.

Name of database		ALS registry Swabia	Emilia Romagna region ALS data	ENCALS	SGH database	TTSH database	SingALS database	University Malaya database	National Institute of Mental Health and Neurosciences database	Peking University database	Taiwan database
Country/region		Germany	Italy	Europe	Singapore	Singapore	Singapore	Malaysia	India	China	Taiwan
Number of patients		663	681	11,475	71	72	143	144	1153	1624	1149
Gender	Male	55.36%	54.48%	/	57.75%	58.30%	58.04%	68.80%	74.20%	63.00%	62.23%
	Female	44.64%	45.52%	/	42.25%	41.70%	41.96%	31.20%	25.80%	37.00%	37.77%
Age of onset (mean/median ± SD/years)		66.6 ± 11.6	66.50 ± 11.09	63.3 (median, 95% CI 54.8–70.7)	57.36 ± 10.57	55.02 ± 11.56	56.18 ± 11.10	57.2 ± 10.9	46.2 ± 14.1	49.8 (median, 95% CI 49.2–50.3)	56.27 ± 14.15
Diagnose delay from symptom onset (mean/median ± SD/months)		6.8 ± 6.1	13.16 ± 13.09	10.5 (median, 95% CI 6.3–17.6)	9.26 ± 7.11	14.44 ± 14.35	11.87 ± 11.60	12 (median)	17.7 ± 20.7	20.2	/
Location of onset (percentage)	Bulbar	34.10%	30.98%	32.00%	26.76%	15.30%	20.98%	28.80%	27.10%	/	/
	Limb	65.90%	69.02%	68.00%	73.24%	84.70%	79.02%	71.20%	72.90%	75.10%	/
Time from symptom onset to clinical milestones (mean/median ± SD/months)	NIV	/	/	/	30.48 ± 15.09	35.46 ± 21.46	33.07 ± 18.66	27 (median)	/	/	/
	PEG	/	/	/	31.82 ± 15.77	33.59 ± 20.81	32.44 ± 17.53	24 (median)	/	/	/
	Disease duration	31.0 ± 0.72	40 (median, 95% CI 36–44)	34.7 (median, 95% CI 34.2–35.3)	44.20 ± 25.88	62.12 ± 41.68	50.51 ± 33.17 40.69 ± 18.76 (2nd and 3rd quartile) 41.17 (median, 95% CI 35.37–46.63)	30 (median)	114.83 ± 25.9	71 (median)	67.75 (median, 95% CI 64.50–70.99)
Riluzole taken (percentage)		/	84.14%	/	14.08%	65.28%	39.86%	23.90%	0.00%	32.30%	69.10%

*Note*: CI, confidence interval; /, lack of data from the study.

For the definition of disease duration, some registries, such as ENCALS [2] and Italy [12] ALS registries, utilize tracheostomy‐free survival as their composite endpoint. Others, including those from Germany [11], Malaysia [15], India [14], China [13], and Taiwan [16], use survival alone as the primary endpoint. By incorporating tracheostomy into our composite endpoint, we ensure better comparability with registries that recognize this significant intervention, while also addressing regional differences in clinical practices and decision‐making frameworks.

### 2.3 Analysis Within the TTSH ALS Registry

#### 2.3.1 Comparative Analysis

Focusing on the potential predictive roles of gender [17], ethnicity [18], and disease progression [19] on survival outcomes, we conducted a detailed comparative analysis of demographic and clinical parameters across different groups within the TTSH ALS database, which represents a more comprehensive and recent clinical cohort. The SGH cohort was excluded from this comparative analysis due to variations in data availability, particularly regarding longitudinal follow‐up and certain key laboratory parameters necessary for our predictive modeling. The categorization into progression groups was based on the slope of the Patient ALS Functional Rating Scale‐Revised (ALSFRS‐R) score [20]. Progression rates were longitudinally tracked using linear regression and classified as slow (absolute value of slope ≤ 0.5/month), intermediate (absolute value of slope between 0.5 and 1.5/month), or fast (absolute value of slope ≥ 1.5/month), in accordance with the criteria from previous studies [7, 21, 22] involving ALS disease progression. In total, 50 patients with sufficient ALSFRS‐R score data were included in the analysis in Table 2. Meanwhile, mortality groups were segregated based on 24‐month survival outcomes since disease onset. In this study, we define mortality as the occurrence of either tracheostomy or death, whichever occurs first.

**TABLE 2:** Comparison of patient demographic and clinical features by gender in TTSH database.

Gender			Total	Male	Female	*p*
All patients: number			72	42	30	
Patients in each ethnicity group: number (percentage)	Chinese		53 (73.6%)	27 (64.3%)	26 (86.7%)	0.034*
	Non‐Chinese	Indian	12 (16.7%)	9 (21.4%)	3 (10.0%)	
		Malay	5 (6.9%)	4 (9.5%)	1 (3.3%)	
		Caucasian	1 (1.4%)	1 (2.4%)	0	
		Eurasian	1 (1.4%)	1 (2.4%)	0	
Location of onset: number (percentage)	Bulbar		11 (15.3%)	6 (14.3%)	5 (16.7%)	0.782
	Limb		61 (84.7%)	36 (85.7%)	25 (83.3%)	
Age of onset (mean ± SD/years)			55.02 ± 11.56	54.61 ± 12.49	55.58 ± 10.29	0.720
Diagnostic delay (mean ± SD/months)			14.44 ± 14.35	11.96 ± 11.62	17.90 ± 17.08	0.105
Time to clinical milestones (mean ± SD/months)	Bedbound		53.45 ± 36.52	45.34 ± 29.83	80.47 ± 50.72	0.354
	NIV		35.46 ± 21.46	29.89 ± 17.08	43.82 ± 25.40	0.150
	PEG		33.59 ± 20.81	26.72 ± 10.46	54.21 ± 31.07	0.119
	Tracheostomy‐free survival		53.92 ± 41.40	46.07 ± 37.30	72.23 ± 40.45	0.119
Medications taken: number (percentage)	Riluzole		47 (65.3%)	27 (64.3%)	20 (66.7%)	
	Edaravone		19 (26.4%)	10 (23.8%)	9 (30.0%)	
	Both drugs taken		18 (25.0%)	9 (21.4%)	9 (30.0%)	

* *p*‐value < 0.006 (Bonferroni adjusted).

Categorical variables were quantified as counts and percentages, while continuous variables were expressed as means ± standard deviations. Differences in continuous variables between gender and mortality groups were analyzed using an unpaired two‐tailed Student's *t*‐test, while differences among progression and ethnicity groups were assessed using Analysis of Variance (ANOVA). For categorical variables, Chi‐square analysis was conducted across the same groups. Bonferroni adjustment for a *p*‐value threshold of 0.05 was applied to minimize false positives during statistical analysis.

All the statistical analyzes were conducted using RStudio 2023.09.0 + 463, available at https://posit.co/products/open‐source/rstudio/.

#### 2.3.2 Survival Analysis

**FIGURE 1:**
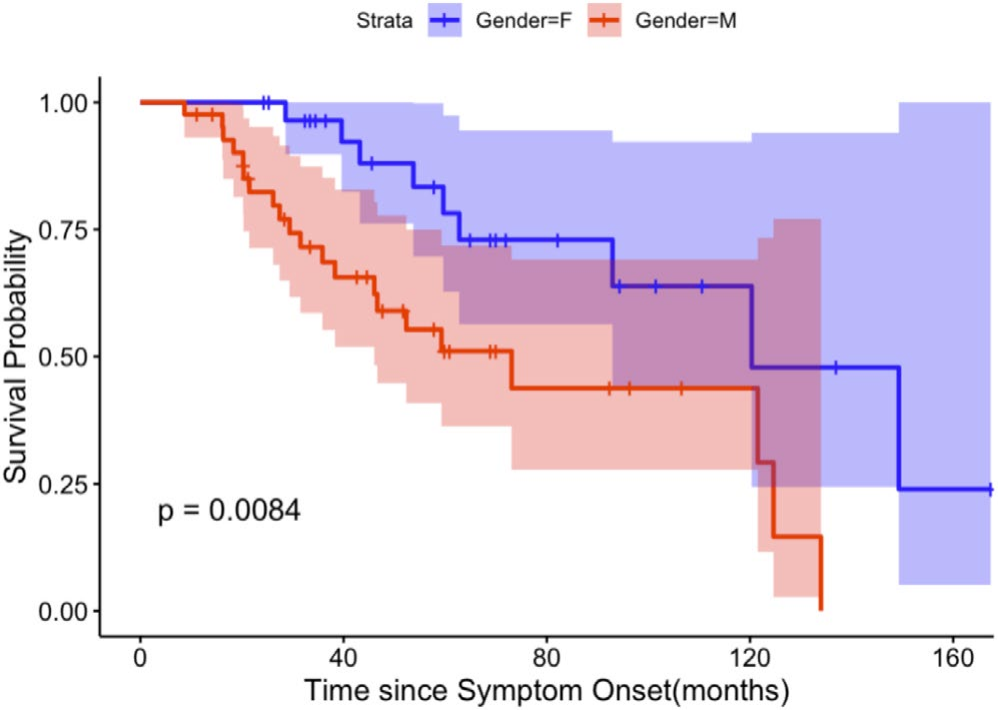
Kaplan–Meier tracheostomy‐free survival plot by gender in TTSH database; *p*‐value is from Log‐Rank test.

We further utilized Kaplan–Meier [23] (KM) survival analysis to chart survival trajectories among different gender and progression groups, illustrated in Figures 1 and S2. Within this framework, an “event” was defined as mortality, with time measured in months from the onset of symptoms to the event. The significance of differences observed in survival across groups was assessed using the Log‐Rank test [23], with each Kaplan–Meier curve's *p*‐value indicating the level of statistical significance.

We then employed univariate Cox regression [24] survival analysis to further investigate the HR associated with each feature. This analysis encompassed demographic and clinical parameters listed in Table 5. Significant predictors, as defined by a *p*‐value of less than 0.050 in the univariate Cox regression analysis, are exclusively listed in Table 4. To further address the confounding factors associated with each variable, we performed a Multiple Cox regression analysis on survival data. The variables were chosen based on their significance and relevance identified in the preceding univariate survival analysis. Additionally, the number of features was selected based on the number of events (mortality) observed in the study. With approximately 30 patient deaths/tracheostomy during the follow‐up period, we included three features in the analysis to ensure an appropriate event‐to‐variable ratio.

All analyzes pertaining to survival were performed utilizing the “survival” package in R.

#### 2.3.3 Machine Learning Analysis

We utilized XGBoost [8] to predict 36‐, 24‐, 12‐, and 6‐month mortality from any clinical visit point using features from the TTSH ALS database. Features used in the model include age, gender, days since onset of symptoms, days since diagnosis, site of onset, Riluzole usage, Edaravone usage, height from 1st visit, weight from 1st visit, full vital capacity (FVC), pulse, ALSFRS‐R, white blood cell count (WBC), red blood cell count (RBC), platelets, absolute neutrophil count, absolute eosinophil count, absolute basophil count, creatinine, albumin, total bilirubin, aspartate transaminase (AST), alanine transaminase (ALT), alkaline phosphatase (ALP), creatinine kinase, bicarbonate, chloride, phosphorus, HbA1c, and protein. All features in our registry were recorded with corresponding timestamps. For training the machine learning model, we treated the features from each specific timepoint as a separate clinical visit. In addition, the time of the measurements was included in the models, such as time from onset of symptoms and time from diagnosis, reflecting the progression of the disease. This approach enables the model to provide mortality predictions based on data from a single visit at any given timepoint, enhancing its clinical applicability. The XGBoost algorithm was implemented using the python package XGBoost. We used a 5‐fold cross‐validation strategy for training and validation within the Singapore dataset. The performance of the trained XGBoost models was assessed using Area Under the Receiver Operator Characteristics Curve (AUC), Area Under the Precision‐Recall Curve (AUPRC), accuracy, sensitivity, specificity, F1 score, positive predictive value (PPV), and negative predictive value (NPV).

The assessment of feature importance within the XGBoost models was determined by the gain each feature provided, aggregating gains across all models from the 5 cross validation folds. This analysis was conducted for the ALS mortality prediction models, considering both the cumulative gain and how frequently a feature appeared among the top 15 influencers to determine its overall importance. In addition, Spearman's correlation was conducted between each influencer and lifespan in days, and this includes both categorical and numerical influencers.

#### 2.3.4 Temporal Dynamics Analysis

To delineate the temporal dynamics of features within distinct mortality groups—specifically, those who died within 24 months versus those who survived beyond this period—we selectively analyzed the top indicators demonstrating temporal variation. These indicators were extracted from the top 15 features outlined by the XGBoost models for 24‐month mortality prediction, as presented in Table S5. To further analyze the relationship between mortality and selected features with a temporal component, we chose 24 months (near midterm mortality) as the marker to split the patients into two mortality groups, due to the limited number of patients. We divided the temporal data into intervals of 10 months for analysis, calculating the mean and standard deviation. Using an unpaired two‐tailed Student's *t*‐test, we also show statistically significant *p*‐values for each subgroup within these intervals.

The temporal feature analysis plot was drawn using the “ggplot2” package in R.

## 3 Results

### 3.1 Comparison of SingALS Database With International Databases

We first compared the several clinical characteristics of the Singapore ALS databases with other ALS registries from Asia and Europe.

Across international ALS registries, the disease shows a male predominance, a trend well documented in previous studies [25]. Our statistical analysis and machine learning models further confirm this pattern within the Singaporean ALS cohort, reinforcing the consistency of gender disparities in ALS incidence. However, diagnostic delays vary widely without a consistent pattern. Bulbar onset remains the minority globally, but Asian patients (from Malaysia [15], India [14], China [13], Taiwan [16] and SingALS) exhibit a lower prevalence of bulbar onset (14.0%–28.8%) compared to European counterparts (32.0%–34.1% in ENCALS [2], Italy [12], and Germany [11]). Additionally, Asian patients show a younger mean or median age of onset (46.2–57.2 years) than Europeans, where the age of onset typically exceeds 60 years. The mean or median time from disease onset to death is also longer in most Asian registries (50.51–114.83 months), with the exception of Malaysia [15] (30 months), compared to European registries (31.0–40.0 months) (Table 1).

### 3.2 Analysis of the Comprehensive TTSH Database

As comprehensive data was collected only in the TTSH ALS cohort, we used this database to examine key demographics and clinical features using statistics and machine learning.

#### 3.2.1 Male Patients Have Worse Prognosis

In the TTSH ALS database, 72 patients were recorded, predominantly of Chinese and Indian ethnicities, male gender, with the majority presenting with limb‐onset as shown in Table 2. Males progressed to major clinical milestones—such as PEG insertion, tracheostomy, and tracheostomy/death—faster (in about half the time) compared to females, despite a similar distribution of known prognostic indicators such as bulbar onset, age of onset, and diagnostic delay [2]. Although these differences in progression between genders were not statistically significant, KM survival analysis showed poorer survival for male patients compared to female patients (*p* = 0.0084) in Figure 1.

#### 3.2.2 Faster ALS Progression Throughout the Disease Worsens Prognosis

KM analysis further demonstrated that patients in the slow progression group experienced a slower progression to mortality in Figure S1 (*p* < 0.0001) and Table 3 (*p* < 0.001).

**TABLE 3:** Comparison of patient demographic and clinical features by speed of progression in TTSH database.

Progressor		Fast	Intermediate	Slow	*p*
Number of patients	Patients in database	6	26	18	
Gender	Male	5 (83.3%)	15 (57.7%)	6 (33.3%)	0.074
	Female	1 (16.7%)	11 (42.3%)	12 (66.7%)	
Patients in each ethnicity group: number (percentage)	Chinese	5 (83.3%)	24 (92.3%)	16 (88.9%)	0.789
	Non‐Chinese	1 (16.7%)	2 (7.7%)	2 (11.1%)	
Location of onset:	Bulbar	2 (33.3%)	3 (11.5%)	2 (11.1%)	0.347
	Limb	4 (66.7%)	23 (88.5%)	16 (88.9%)	
Age (mean ± SD/years)	Age of Onset	57.12 ± 11.92	54.71 ± 9.10	58.13 ± 11.05	0.538
Diagnose delay (mean ± SD/months)		14.37 ± 20.66	29.70 ± 8.77	19.15 ± 19.35	0.368
Average ALSFRS‐R Slope/month		−2.41 ± 0.88	−1.01 ± 0.29	−0.22 ± 0.16	< 0.001*
Time to clinical milestones (mean ± SD/months):	NIV	7.1	35.84 ± 15.22	55.79 ± 30.38	NA
	PEG	15.7 ± 0.66	31.97 ± 10.43	68.52 ± 58.10	0.035
	Tracheostomy‐free survival	37.6 ± 31.07	43.54 ± 15.98	129.95 ± 12.06	< 0.001*
Medications taken	Riluzole	5 (83.3%)	19 (73.1%)	10 (55.6%)	
	Edaravone	3 (50%)	9 (34.6%)	4 (22.2%)	
	Both drugs taken	3 (50%)	8 (30.8%)	4 (22.2%)	

*Note*: NA: lack of sufficient data in one or more subgroups to calculate *p* value.

* *p*‐value < 0.006 (Bonferroni adjusted).

#### 3.2.3 Features of ALS Patients With Poorer Mortality

ALS patients who needed tracheostomy or died within 24 months had a significantly quicker need for being bedbound (*p* = 0.004), PEG insertion (*p* = 0.004) and NIV (*p* < 0.001) compared to their counterparts who survived 24 or more months, as shown in Table 4.

**TABLE 4:** Comparison of patient demographic and clinical features by 24‐month mortality in TTSH database.

Mortality		Survival within 24‐month period	Mortality within 24‐month period	*p*
Number of patients		65	7	
Number (percentage) of patients in each ethnicity group	Chinese	50 (76.9%)	3 (42.9%)	0.052
	Non‐Chinese	15 (23.1%)	4 (57.1%)	
Location of onset	Bulbar	8 (12.3%)	3 (42.9%)	0.033
	Limb	57 (87.7%)	4 (57.1%)	
Age of onset (mean ± SD/years)		54.37 ± 11.51	60.98 ± 10.98	0.173
Diagnostic delay (mean ± SD/months)		15.00 ± 14.80	9.22 ± 7.95	0.128
Time to clinical milestones (mean ± SD/months)	Bedbound	59.65 ± 36.40	19.32 ± 1.39	0.004*
	NIV	37.66 ± 20.94	10.15 ± 4.31	< 0.001*
	PEG	35.35 ± 21.23	17.72 ± 2.19	0.004*
Number of patients medications taken (percentage)	Riluzole	41 (63.1%)	6 (85.7%)	
	Edaravone	18 (27.7%)	1 (14.3%)	
	Both drugs taken	17 (26.2%)	1 (14.3%)	

*Note*: NA: lack of sufficient data in one or more subgroups to calculate *p* value.

* *p*‐value < 0.007 (Bonferroni adjusted).

#### 3.2.4 Cox Regression Survival Analysis

While Kaplan–Meier analysis is useful for visualizing and comparing survival data, Cox regression is essential for evaluating the impact of multiple variables on survival and adjusting for confounders. Initially, univariate Cox regression analysis showed male ALS patients have a higher risk of death compared to females (*p* = 0.012); similarly, shorter time taken for a patient to become bedbound is associated with poorer survival outcomes (*p* = 0.017) (Table 5).

**TABLE 5:** Univariate Cox regression analysis of variables affecting ALS tracheostomy‐free survival in TTSH database.

Variables significantly affecting survival	Total (*N* = 72)	HR	95%CI	*p*
Gender: Female (%)	41.67	Reference	Reference	Reference
Gender: Male (%)	58.33	2.88	1.27, 6.55	0.012*
Time to from symptom onset to bedbound (months)	53.45 ± 36.52	0.82	0.69, 0.96	0.017*
Onset age (years)	55.78 ± 11.72	1.03	0.99, 1.06	0.114
Diagnostic delay (years)	14.44 ± 14.35	0.98	0.96, 1.01	0.180
Bulbar onset (%)	15.28	Reference	Reference	Reference
Limb onset (%)	84.72	0.57	0.24, 1.37	0.209
Time to from symptom onset to tracheostomy (months)	30.06 ± 11.89	< 0.001	< 0.001, > 999	0.514
Time to from symptom onset to wheelchair bound (months)	31.94 ± 25.76	0.99	0.97, 1.01	0.207
Riluzole use (%)	65.28	1.63	0.76, 3.50	0.211
Edaravone use (%)	26.39	0.87	0.33, 2.34	0.788

* *p*‐value < 0.050.

Multivariate Cox regression was then further conducted, showing male patients exhibit worse survival outcomes compared to female patients (*p* = 0.008) after adjusting for all the parameters in Table 6.

**TABLE 6:** Multiple cox regression analysis for variables contributing to tracheostomy‐free survival in TTSH database.

Variables	aHR	95%CI	*p*
Gender: Male	3.12	1.35, 7.24	0.008*
Limb onset	0.45	0.18, 1.12	0.085
Onset age	1.00	0.99, 1.01	0.167

* *p*‐value < 0.050.

#### 3.2.5 Top Predictive Features Identified by Machine Learning Analysis

Machine learning analysis can approximate complex relationships within the data without needing to explicitly understand the underlying functions, using all the features including the laboratory features and looking at mortality from any clinical visit time point. Utilizing features shown in Table S1, the XGBoost machine learning model showed greater predictive AUC for short‐term (6‐months) mortality over long‐term (24‐months) mortality (Table S3).

The analysis of the top 15 mortality prediction features from the XGBoost models across different predictive windows identified multiple features including clinical features, such as age, diagnostic delay, and sex, and laboratory tests related to inflammation, such as WBC count, absolute neutrophil count, and ALP. Other significant features are listed in descending order in Table S4 and shown in Figure S2.

Using Spearman's correlation analysis, higher values of WBC count, absolute neutrophil count, and ALP are negatively correlated with lifespan, whereas the converse is true for creatinine and ALSFRS‐R slope (Table S6).

#### 3.2.6 Temporal Feature Analysis Showed Dysregulation of Selected Predictive Features in the Initial 20 Months Following Symptoms Onset

**FIGURE 2:**
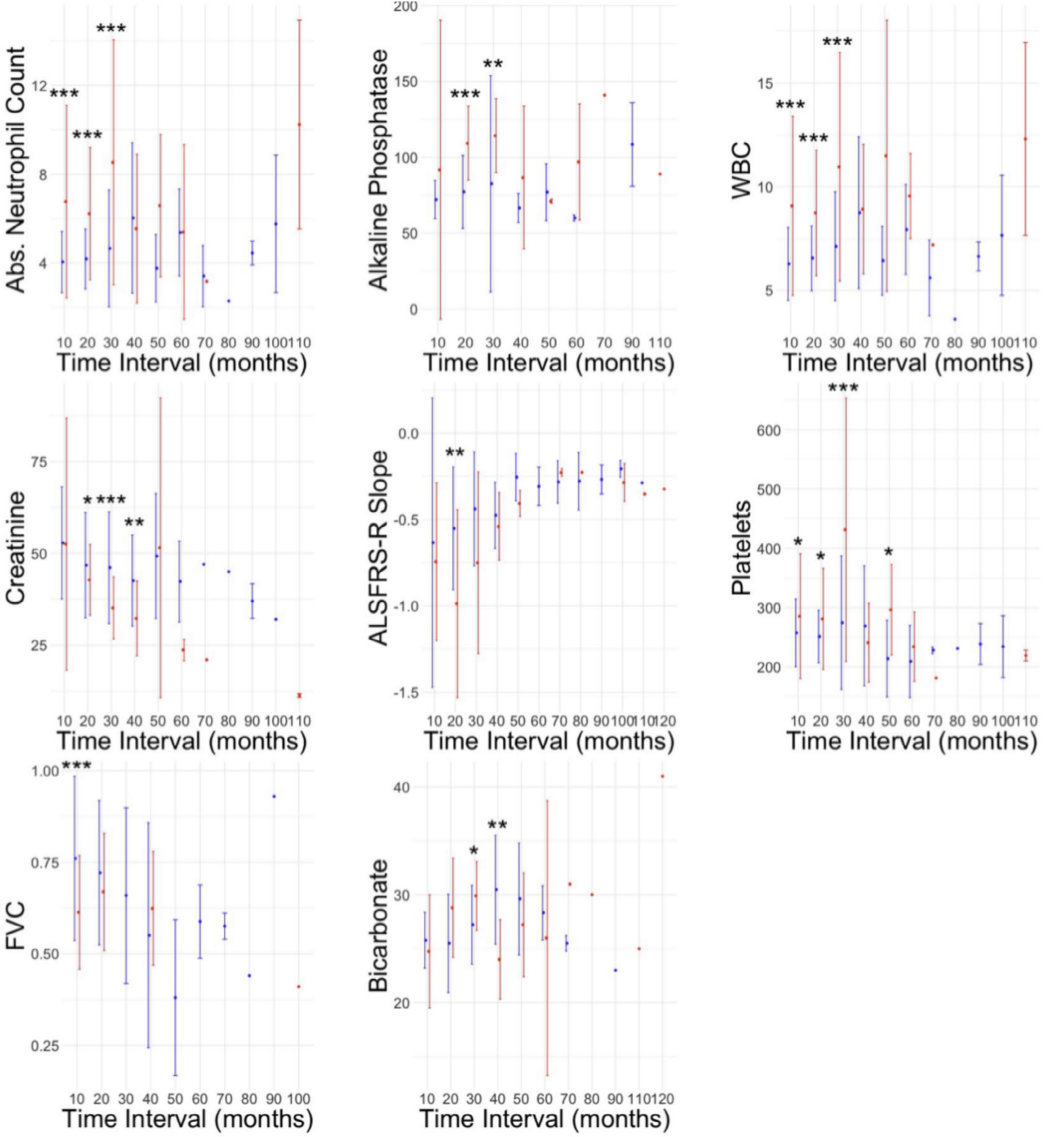
Mean and standard deviation bar plot based on the top features with temporal components selected by the machine learning model in TTSH database. Survival outcome comparison between patients who died or received tracheostomy within 24 months (red) and those who did not die or receive tracheostomy (blue). *p value < 0.050, **p value < 0.010, ***p value < 0.001. Dot: Mean value; Bar: Standard deviation.

When analyzed over the entire time‐course of disease until the last collection of laboratory samples, patients who died within 24 months of ALS onset have a higher WBC count, neutrophil count, and platelet levels during the initial 20 months following symptom onset (Figure 2).

Similarly, across the entire disease course, male patients demonstrated higher WBC counts, neutrophil counts, ALSFRS‐R decline, and creatinine levels during the initial 20 months after symptom onset (Figure S3).

## 4 Discussion

### 4.1 Comparison Among the International ALS Databases

Similar to ALS patients from Asian databases [6, 13, 14, 15, 16], the Singaporean ALS cohort has a longer disease duration compared to the patients from Caucasian‐predominant databases [11, 26]. This cohort has a lower incidence of bulbar onset ALS, a known predictor of poorer survival [27], and a relatively younger age of onset of ALS. These findings underscore the geographical differences in ALS clinical presentations and prognoses, which could be influenced by ethnic diversity [28].

Of note too is the disparity in treatment accessibility across Asia. Our study shows that Singapore has a relatively higher percentage of patients receiving Riluzole, while lower‐income countries, such as India, show a significantly reduced or even negligible percentage of patients with access to this medication [29]. This highlights the ethical need for future studies to explore the impact of financial burden and cultural acceptance on ALS treatment outcomes.

### 4.2 Role of Gender on ALS Survival Outcomes

Previous research on the role of gender in ALS survival outcomes has yielded mixed results. Studies focusing on predominantly Caucasian populations have reported shorter survival prognoses for female patients, attributing this trend, in part, to a higher incidence of bulbar onset among women in an Italian ALS cohort [17]. Conversely, research within predominantly Chinese cohorts, similar to the Singaporean cohort, suggests that female patients tend to have better survival outcomes [13]. Low BMI [30, 31, 32] and adiposity [19] have been shown to reduce ALS survival. In men, more rapid weight loss and worsening of lung function have been linked to shorter survival time [33]. In the TTSH cohort, however, gender has no impact on BMI (*p* = 0.07) at the first visit. Furthermore, temporal analysis shows that gender had no impact on changes in FVC throughout disease progression. However, we have found that the inflammatory response in male and female patients is significantly different, where higher WBC and neutrophil counts in male patients correlate with worse survival outcomes (Figure S3).

### 4.3 Using ALSFRS‐R Slope to Predict ALS Survival Outcomes

The ALSFRS‐R slope has been established as a prognostic biomarker for ALS survival, with a faster initial decline associated with poorer outcomes [19, 33]. However, the progression of ALSFRS‐R scores has been observed to follow a curvilinear pattern, with a more rapid decline in the early and late stages of the disease, while the mid‐stage exhibits a slower rate of deterioration [34]. Although changes in ALSFRS‐R scores reflect functional decline, they do not always correspond directly to the timing of clinical milestones, as patients may be at different stages of disease progression.

Our study corroborates these findings, demonstrating that fast progressors throughout the period of follow‐ups have shorter survival times and more rapid progression to death or tracheostomy (Table 3). This is further supported by both machine learning models and Spearman's correlation analysis, showing steeper ALSFRS‐R slopes as one of the key predictors of poorer ALS mortality in Singaporean ALS patients (Tables S4 and S6). This role of ALSFRS‐R slope as a predictor of ALS mortality in our study is consistent with prior research [19, 33].

### 4.4 Using Inflammatory Markers to Predict ALS Survival Outcomes

Our study also identified other predictors of poorer ALS mortality in Singaporean ALS patients, supported by both machine learning models and Spearman's correlation analysis. These predictors include higher ALP, elevated WBC and neutrophil counts. Temporal feature analysis further confirmed that patients who died within 24 months of ALS onset typically exhibited higher WBC count and increased neutrophil counts early in their disease course (Figure 2). Studies have shown that early treatment of ALS patients provides better ALS outcomes [35, 36]. Recognizing these early markers can enable more proactive monitoring and early treatment with anti‐inflammatory drugs.

Additionally, elevated baseline neutrophil counts have been linked to higher mortality in ALS patients [37]. ALP, recognized as a marker for predicting functional decline in ALS [38], is an enzyme released from white blood cells and is likely correlated with absolute neutrophil counts [39]. This finding supports existing research indicating that an enhanced immune response, observed in both Caucasian and Asian patients [40], may influence ALS progression. Gendelman's [41] work on T cell regulation and the potential anti‐inflammatory effects of Masitinib on mast cells [42] also suggests immune modulation can be a potential therapeutic pathway.

### 4.5 Limitations and Future Directions

International comparisons are challenging due to variations in healthcare costs, availability of specialized services, and cultural differences in healthcare‐seeking behavior [43]. Additionally, this study is limited by the small sample size, with 72 patients in the TTSH database and 143 patients in the SingALS database, which confines the ability to draw broad conclusions. Although small, this cohort represents a significant proportion—more than 58.8%—of all ALS cases in Singapore, considering that only 243 motor neuron disease cases were reported in 2016, and is highly representative of the ALS population in Singapore. Therefore, our study, while limited, captures key clinical and demographic features necessary for meaningful analysis. Our research involved developing and evaluating a machine learning model to predict ALS mortality within specific time frames. A more comprehensive and balanced patient dataset, enriched with a wider range of ALS mortality cases, can enhance the model's predictive capabilities in Asian ALS patients. The small sample size may have impacted the significance of certain prognostic factors, such as age and site of onset, in the multivariable Cox regression analysis. However, our machine learning analysis identified these features among the top 5 predictors for mortality. This highlights the added value of the machine learning model in uncovering important predictive features that may not achieve statistical significance in traditional regression models due to small patient cohorts in rare diseases. Larger, international, population‐based studies are needed to validate our findings and further investigate the effects of ethnicity and healthcare systems on patient outcomes. Collecting and analyzing laboratory data over larger multi‐ethnic databases can better identify potential mechanisms underlying ALS pathophysiology and open avenues for targeted interventions.

## Author Contributions

**Crystal Jing Jing Yeo, Savitha Ramasamy:** conceptualization. **Ling Guo, Ian Qian Xu, Jing Xu, Savitha Ramasamy, Crystal Jing Jing Yeo:** methodology. **Ling Guo, Ian Qian Xu, Zachary Simmons, Savitha Ramasamy, Crystal Jing Jing Yeo:** analysis. **Ling Guo, Ian Qian Xu, Zachary Simmons, Savitha Ramasamy, Crystal Jing Jing Yeo:** writing – original draft preparation. **Ling Guo, Ian Qian Xu, Stella Setiawan, Xiao Deng, Yew Long Lo, Josiah Yui Huei Chai, Zachary Simmons, Jing Xu, Savitha Ramasamy, Crystal Jing Jing Yeo:** writing – review and editing. **Crystal Jing Jing Yeo, Savitha Ramasamy:** supervision.

## Ethics Statement

We confirm that we have read the Journal's position on issues involved in ethical publication and affirm that this report is consistent with those guidelines.

## Conflicts of Interest

The authors declare no conflicts of interest.

## Supporting information


**Data S1.** Supporting Information.

## Data Availability

The data that support the findings of this study are available on request from the corresponding author. The data are not publicly available due to privacy or ethical restrictions.
