# Supplementary material for: Predictive Analysis of Amyotrophic Lateral Sclerosis Progression and Mortality in a Clinic Cohort From Singapore
Source: Muscle Nerve. 2025 Apr 23;72(1):71–81. doi: 10.1002/mus.28416 (PMC12138491; doi:10.1002/mus.28416)
Supplement: Supplementary file 1 — Data S1. Supporting Information. [file MUS-72-71-s001.docx]

**SUPPLEMENTARY MATERIALS**

**Supp. Table 1:** All features in the SGH/SingALS Database

| **Category** | **Parameter** | **Units** | **Percentage Missing** |
| --- | --- | --- | --- |
| Demographic | Date of birth | days | 0 |
|  | Sex |  | 0 |
|  | Age | years | 0 |
|  | Ethnicity |  | 0 |
| Clinical | Days since onset of symptoms | days | 0 |
|  | Days since diagnosis | days | 0 |
|  | Site (bulbar/limb) | days | 0 |
|  | Date of tracheostomy | days | 0 |
|  | Date of PEG insertion | days | 0 |
|  | Date of NIV | days | 0 |
|  | Date of wheelchair bound | days | 0 |
|  | Date of bedbound | days | 0 |
|  | Riluzole use |  | 0 |
|  | Edaravone use |  | 0 |

**Supp. Table 2:** All features collected in the TTSH ALS Database

| **Category** | **Parameter** | **Units** | **Percentage Missing** |
| --- | --- | --- | --- |
| Demographic | Date of birth | days | 0 |
|  | Sex |  | 0 |
|  | Age | years | 0 |
|  | Ethnicity |  | 0 |
| Clinical | Days since onset of symptoms | days | 0 |
|  | Days since diagnosis | days | 0 |
|  | Site (bulbar/limb) | days | 0 |
|  | Date of tracheostomy | days | 0 |
|  | Date of PEG insertion | days | 0 |
|  | Date of NIV | days | 0 |
|  | Date of wheelchair bound | days | 0 |
|  | Date of bedbound | days | 0 |
|  | Progression status |  | 0 |
|  | Riluzole use |  | 0 |
|  | Edaravone use |  | 0 |
| Laboratory | Height from 1^st^ visit | m | 0 |
|  | Weight from 1^st^ visit | kg | 65.3 |
|  | FVC (as % of predicted for normal individual)/3 months | proportion | 77.7 |
|  | Pulse/3 months | bpm | 97.1 |
|  | ALSFRS-R/3 months |  | 51.8 |
|  | WBC | 10E9/L | 47.0 |
|  | RBC | 10E9/L | 47.1 |
|  | Platelets | 10E9/L | 47.0 |
|  | Abs. Neutrophil | 10E9/L | 48.2 |
|  | Abs. Eosinophil | 10E9/L | 47.6 |
|  | Abs. Basophil | 10E9/L | 47.6 |
|  | Creatinine | μmol/L | 43.8 |
|  | Albumin | g/L | 47.9 |
|  | Total Bilirubin/3 months | μmol/L | 45.1 |
|  | AST/3 months | U/L | 32.4 |
|  | ALT/3 months | U/L | 19.2 |
|  | Alkaline Phosphatase/3 months | U/L | 24.4 |
|  | CK/3 months | U/L | 56.3 |
|  | Bicarbonate/3 months | mmol/L | 75.0 |
|  | Chloride/3 months | mmol/L | 95.1 |
|  | Phosphorus/3 months | mmol/L | 85.4 |
|  | HbA1c/3 months | % | 97.5 |
|  | Protein/3 months | g/L | 96.9 |

**Supp. Table 3:** 24 and 6-month mortality* prediction on the TTSH ALS database using all features trained on TTSH database at any timepoint in the disease course

| Model | XGBoost | | |
| --- | --- | --- | --- |
| Prediction months | 24 | 6 | |
| Train Size | 957 ± 78 | | |
| Test Size | 239 ± 78 | | |
| % died | 28.1 | | 7.4 |
| AUC | 0.52 ± 0.119 | | 0.712 ± 0.099 |
| AUPRC | 0.316 ± 0.151 | | 0.214 ± 0.064 |
| Accuracy | 0.599 ± 0.114 | | 0.905 ± 0.042 |
| Sensitivity | 0.276 ± 0.161 | | 0.162 ± 0.117 |
| Specificity | 0.733 ± 0.194 | | 0.963 ± 0.025 |
| F1 | 0.249 ± 0.121 | | 0.176 ± 0.126 |
| PPV | 0.305 ± 0.217 | | 0.200 ± 0.143 |
| NPV | 0.731 ± 0.098 | | 0.936 ± 0.033 |

*Mortality is defined as death or tracheostomy

**Supp. Table 4:** Top 15 features for mortality prediction for XGBOOST models trained across 6-, 12- and 24-months mortality prediction models

| Rank | Feature | Association with higher mortality risk |
| --- | --- | --- |
| 1 | Age | older |
| 2 | Diagnostic Delay | longer |
| 3 | Abs. Neutrophil Count | Higher |
| 4 | BMI | Lower |
| 5 | Alkaline Phosphatase | Higher |
| 6 | Sex | Male |
| 7 | WBC Count | Higher |
| 8 | Creatinine | Lower |
| 9 | Onset Site | Bulbar |
| 10 | ALSFRS-R Slope | Steeper |
| 11 | Platelets | Higher |
| 12 | FVC | Lower |
| 13 | Bicarbonate | Higher |
| 14 | Edaravone Use | No |
| 15 | Riluzole Use | Yes |

**Supp. Table 5:** Features with temporal variation chosen for temporal dynamic analysis

| **Parameter** | **Units** |
| --- | --- |
| ALSFRS-R Slope |  |
| WBC Count | 10E9/L |
| Abs. Neutrophil Count | 10E9/L |
| FVC | proportion |
| Creatinine | μmol/L |
| Bicarbonate | μmol/L |
| Alkaline Phosphatase | U/L |
| Platelets | 10E9/L |

**Supp. Table 6:** Spearman’s correlation between the number of survival days for top 15 predictors identified by TTSH ALS database trained XGBOOST model

| **Predictors** | **Correlation** | **p Value** |
| --- | --- | --- |
| Age | -0.18 | 0.353 |
| Diagnostic Delay | 0.20 | 0.305 |
| Abs. Neutrophil Count | -0.29 | < 0.001* |
| BMI from 1^st^ visit | 0.83 | 0.015 |
| Alkaline Phosphatase | -0.33 | < 0.001* |
| Sex (Male) | -0.10 | 0.033 |
| WBC Count | -0.24 | < 0.001* |
| Creatinine | 0.44 | < 0.001* |
| Bulbar Onset | 0.103 | 0.028 |
| ALSFRS-R Slope | 0.51 | < 0.001* |
| Platelets | -0.04 | 0.560 |
| FVC | 0.28 | 0.025 |
| Bicarbonate | -0.10 | 0.394 |
| Edaravone Use | 0.09 | 0.067 |
| Riluzole Use | -0.03 | 0.474 |

* p value < 0.0033 (Bonferroni adjusted)

**
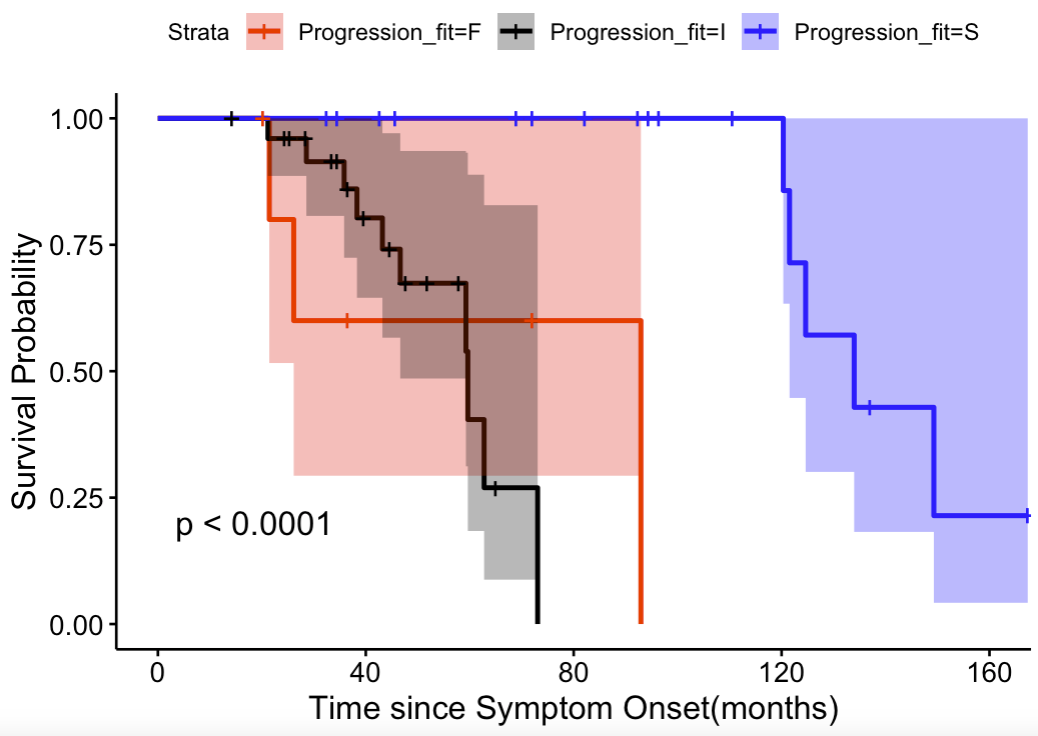
**

**Supp. Figure 1:** Kaplan-Meier tracheostomy-free survival plot by progression in TTSH database, p-value is from Log-Rank test.


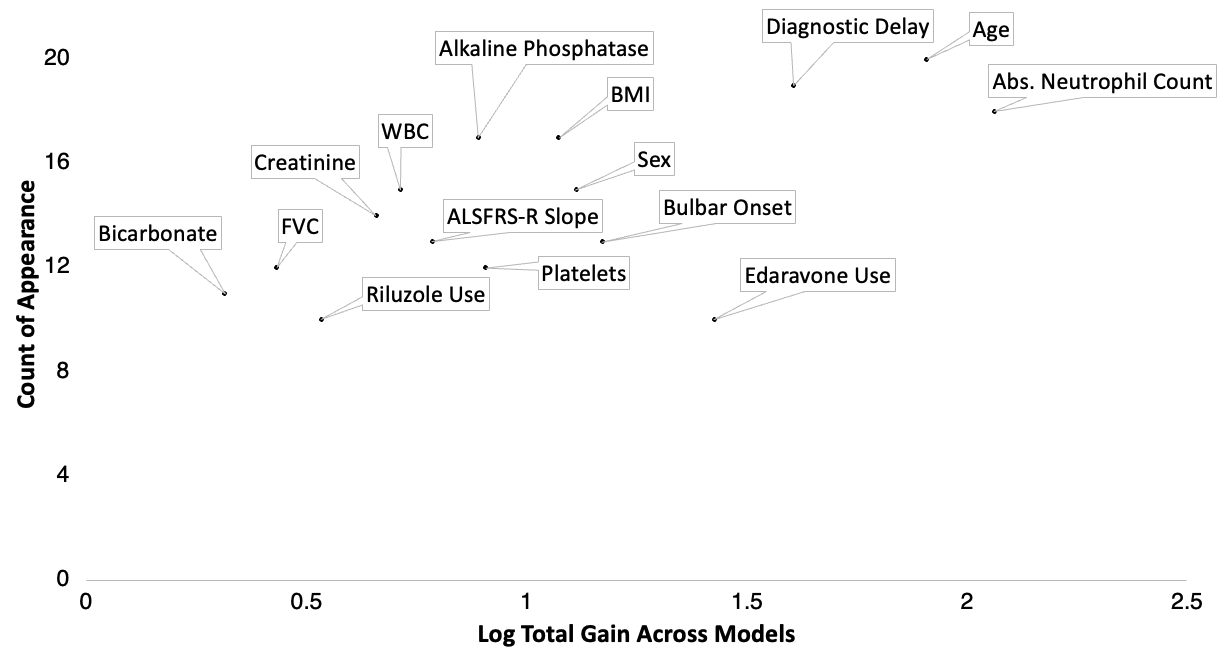


**Supp. Figure 2:** Top 15 features for mortality prediction based on their ranking of count of appearance and log total gain across models. Mortality is defined as death or tracheostomy.


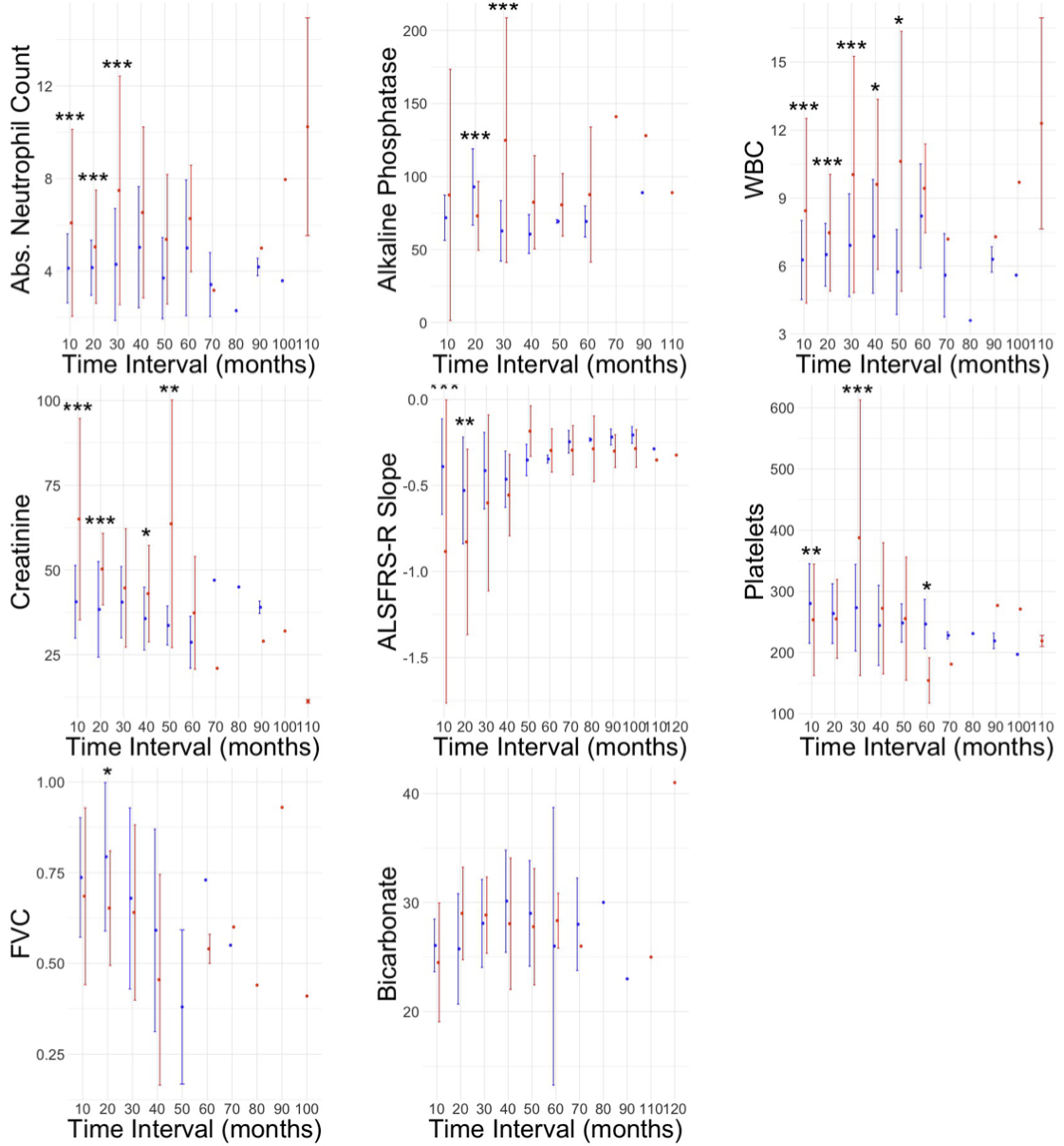


**Supp. Figure 3:** Mean and standard deviation bar plot based on the top features with temporal components selected by the machine learning model between male (red) and female (blue) in TTSH database.

* p value < 0.050, ** p value < 0.010, *** p value < 0.001

Dot: mean value; Bar: standard deviation.
